# The influence of socioeconomic characteristics on active travel in US metropolitan areas and the contribution to health inequity

**DOI:** 10.12688/wellcomeopenres.19147.1

**Published:** 2023-06-21

**Authors:** Samuel Younkin, Henry Fremont, Jennifer Bratburd, Daritza De Los Santos, Jonathan Patz

**Affiliations:** 1 Global Health Institute, University of Wisconsin-Madison, Madison, WI, 53726, USA; 2 Nelson Institute for Environmental Studies, University of Wisconsin-Madison, Madison, WI, 53726, USA; 3 Department of Population Health Sciences, University of Wisconsin-Madison, Madison, WI, 53726, USA

**Keywords:** active transportation, active travel, cycling, bicycling, walking, health inequity

## Abstract

*Background*

The prevalence of chronic disease in the US adult population varies across socioeconomic groups in the USA where approximately six in 10 adults have a chronic condition. Walking or cycling reduces the risk to many of these diseases and is influenced by the built environment, accessibility, and safety.

*Methods*

We performed multivariate logistic and linear regression on the Health-Oriented Transportation model parameters using the 2009 and 2017 US National Household Transportation surveys, restricted to adults in major metropolitan areas. Model covariates included socioeconomic and environmental characteristics.

*Results*

Using odds ratios (OR) adjusted for model covariates, we observe several significant variables in 2009 and 2017. Residents of households with no cars were more likely to walk or cycle than those with two cars; OR=5.4 (4.8, 6.0). Residents of households in a census block with population density greater than 2,5000 persons/square mile were more likely to walk or cycle than those with a population density of 2,000–3,999; OR=2.6 (2.3, 2.8). Individuals with a graduate or professional degree were more likely to walk or cycle than those with a high school degree; OR=2.1 (1.9, 2.2). Individuals that self-report as Black or African American, or Asian are less likely to walk or cycle than White; OR=0.60 (0.56, 0.66), OR=0.70 (0.65, 0.75). The proportional increase in all-cause mortality from estimated reductions in physical activity for African American, Asian, and Hispanic populations were 1.0%, 0.7%, 0.8%, respectively.

*Conclusions*

Access to automobiles and the surrounding population density are primary factors in the decision to walk or cycle. After adjusting for these and other factors, members of low-income, low-education, Black or African American, and Asian populations in US metropolitan areas are less likely to walk or cycle than high-income, high-education, or White populations and the discrepancy in physical activity is likely to contribute to health inequity.

## Introduction

Currently in the USA approximately six in 10 adults have a chronic condition such as heart disease, stroke, cancer, or diabetes ( US Center for Disease Control and Prevention). In 2020 seven of the top 10 causes of death were chronic diseases ^1^. Disparities in the prevalence of chronic disease across race and ethnicity is a problem faced by the United States with some studies suggesting there that has been no improvement in the last 20 years ^2– 4^. Risk factors for chronic disease are known to include tobacco use, poor diet and physical inactivity ^5^. Here we focus on the physical inactivity risk factor for chronic disease and examine how socioeconomic factors affect transportation-related physical activity and the corresponding potential health inequity.

Studies have shown that commuting by walking or cycling provides sufficient physical activity to reduce the risk of some chronic disease ^6, 7^. A meta-analysis of commuting by walking or cycling and cardiovascular disease reported an 11% reduction in cardiovascular risk (RR=0.89) in those who commute by walking or cycling compared to those who do not ^6^. In 2009 and 2017, the percentage of adult Americans meeting the minimum aerobic physical activity guidelines published by the US Center for Disease Control and Prevention (>150 minutes of moderate-intensity exercise per week) was only 47.2% (46.2%–48.2%) and 54.1% (52.9%–55.2%), respectively ^8^.

Participation in transportation-related walking or cycling (active travel) is known to be influenced by the built environment and access to public transportation ^9, 10^. Less is known, however, about which sociodemographic characteristics are associated with active travel, after controlling for known environmental factors. Using the National Household Transportation Survey data from 2009 and 2017, we identified clear disparities in active travel across race, education, and household income in major US metropolitan areas in both 2009 and 2017 which have not been previously reported in the literature. Our analysis is the first to restrict the sample to major metropolitan areas, and one of the few to use regression models with social and environmental covariates. We use the Health-Oriented Transportation (HOT) model to estimate how these disparities may affect the health of US subpopulations ^11^.

## Methods

### Data Description

We used publicly available US National Household Travel Survey data from 2009 and 2017 distributed by the US Department of Transportation, Federal Highway Administration. The 2009 survey is a one-day, list-assisted, stratified, random-digit dialing of households with landline telephones with a sample size of 150,147 households ^12– 14^. The 2017 survey is also a one-day survey, but is the combination of an address-based national sample of households with 13 add-on statewide samples purchased by various states/Metropolitan Planning Organizations (MPOs) for a total of 129,696 households (Arizona; California; Dallas-Fort Worth, Texas; Des Moines, Iowa; Georgia; Maryland; New York; North Carolina; South Carolina; Texas; Tulsa, Oklahoma; Waterloo, Iowa; and Wisconsin) ^14^. We restricted the sample to adults aged 19 to 65 years old. The prevalence of active travel in non-urban areas of the USA is significantly less than in urban areas so we further restricted our analysis to households in metropolitan areas with a population size greater than one million ^12^. The age cutoffs and urban area restriction left 58.6% and 60.9% of the sampled subjects and 44.5% and 43.1% of the sampled households in 2009 and 2017, respectively. Subjects with missing data needed for participation estimates were removed (0.8% for 2009, 0.3% for 2017). Data from any state with a sample size of less than 150 households (1370 for 2009, and 912 households for 2017) and from any subject with outlying travel activity estimates (>50 metabolic equivalent unit [MET]-hours/week) were excluded from analysis (15 subjects in 2009 and 29 subjects in 2017). MET represents the energy expenditure of a physical activity as the ratio of an individual’s metabolic rate while performing the physical activity to a standardized resting metabolic rate of approximately 3.5 ml O _2_/kg/min ^15, 16^. The data filtering resulted in a final dataset of 47,819 and 41,761 households, in which 81,226 and 72,345 adults resided in 2009 and 2017, respectively.

Values for the race variable were recorded as the respondent’s choice from among a predefined list defined by the survey designers. In 2009, the choices were “White”, “African American, Black”, “Asian”, “American Indian, Alaskan Native”, “Native Hawaiian, or other Pacific Islander”, “Multiracial”, “Hispanic/Mexican”, “other”, “refused”, and “don’t know.” Respondents were asked to report which one best describes their race. In 2017, the choices were “White”, “Black or African American”, “Asian”, “American Indian or Alaskan native”, “Native Hawaiian or other Pacific islander”, “Some other race”, “I don’t know”, and “I prefer not to answer.” The respondent was asked to select all that apply. Individuals that selected multiple values in 2017 along with respondents who selected “Multiracial” in 2009 are identified in the data as “Multiple Responses Selected.” In 2009, only the household respondent was asked to report their race. This value was used for all members of the household. Because “Hispanic/Mexican” was not an option in 2017, respondents who chose “Hispanic/Mexican” in 2009 were grouped with “other.” See the Limitations section for more discussion on the category labels.

Table 1 provides summary statistics for selected social and environment variables in the sample. Social variables include sex, age, race, Hispanic status, race by Hispanic status, household income, and education. The environmental variables included in Table 1 are the discretized population density for the household’s census block, and the number of cars per household (0, 1, 2, 3+). Values for race were recorded as the respondent’s choice from among a predefined list. The Hispanic variable was coded as true if the respondent identified as being of “Hispanic or Latino Origin” in 2017, or of having “Hispanic status” in 2009. In 2009 both the race and Hispanic variables were recorded only for the household respondent and used for each person in the household.

**Table 1.** The sample size of the US National Household Travel Surveys from 2009 and 2017 restricted to adults living in a US metropolitan area with a population size greater than one million and stratified by each of the social and environmental variables considered here.

	National Household Travel Survey	
Characteristic	2009, N = 81,226 (53%)	2017, N = 72,345 (47%)
Sex, n (%)		
Female	43,948 (54)	38,271 (53)
Male	37,278 (46)	34,014 (47)
NA	0	60
Race, n (%)		
American Indian or Alaska Native	644 (0.8)	330 (0.5)
Asian	3637 (4.5)	6292 (8.8)
Black or African American	5315 (6.6)	6038 (8.4)
Multiple responses selected	707 (0.9)	2222 (3.1)
Native Hawaiian or other Pacific Islander	392 (0.5)	250 (0.3)
Other	4977 (6.2)	2383 (3.3)
White	64,652 (80)	54,364 (76)
NA	902	466
Hispanic, n (%)		
No, not Hispanic or Latino	71,561 (88)	63,604 (88)
Yes, Hispanic or Latino	9301 (12)	8652 (12)
NA	364	89
Household income, n (%)		
Less than USD 1,0000	2387 (3.1)	2233 (3.2)
USD 1,0000 to 1,4999	1853 (2.4)	1794 (2.5)
USD 1,5000 to 2,4999	4111 (5.4)	3372 (4.8)
USD 2,5000 to 34,999	4899 (6.4)	3965 (5.6)
USD 3,5000 to 4,9999	8986 (12)	5940 (8.4)
USD 5,0000 to 7,4999	13,390 (17)	10,679 (15)
USD 7,5000 to 9,9999	13,720 (18)	10,422 (15)
USD 10,0000 or more	27,228 (36)	32,310 (46)
NA	4652	1630
Education, n (%)		
Less than a high school graduate	3588 (4.5)	1837 (2.5)
High school graduate or GED	16,457 (20)	10,310 (14)
Some college or associates degree	23,165 (29)	19,646 (27)
Bachelor's degree	21,765 (27)	22,311 (31)
Graduate degree or professional degree	15,510 (19)	18,207 (25)
NA	741	34
Population density, n (%)		
0-99	3410 (4.2)	1968 (2.7)
100-499	7962 (9.8)	6241 (8.6)
500-999	5735 (7.1)	4543 (6.3)
1000-1999	10,329 (13)	8331 (12)
2,000-3,999	18,171 (22)	15,196 (21)
4000-9999	25,531 (31)	24,671 (34)
1,0000-2,4999	7567 (9.3)	8295 (11)
2,5000-99,9999	2521 (3.1)	3096 (4.3)
NA	0	4
Cars per household, n (%)		
0	2357 (2.9)	2591 (3.6)
1	11,798 (15)	14,549 (20)
2	36,936 (45)	31,251 (43)
3+	30,135 (37)	23,954 (33)
NA	0	0

Trips in the 2017 survey with the stated purpose *Exercise (go for a jog, walk, walk the dog, go to the gym)*, and trips in the 2009 survey with the purpose *Go to gym/exercise/play sports* were removed from the analysis (3.4% and 3.9% of all trips, respectively) since unrelated to travel. Trips with no mode of travel listed were removed from analysis (0.2%, 0.002% for 2009, 2017). 

### Prevalence and participation

As in Younkin *et al.* (2021), we made a distinction between prevalence and participation in active travel ^11^. The prevalence of active travel is a one-day snapshot of the proportion of active travelers, while participation is the proportion of active travelers over one week. An active traveler is defined as a respondent that reported either a walk or cycle trip on the survey day. We estimated 95% confidence intervals for the prevalence of active travel among adults in US metropolitan areas, stratified by social and environmental variables. We used the sample weights provided in the NHTS data set. Two self-reported values for the number of trips taken by walking and cycling over the past week are included in the NHTS data and we use these to estimate participation as the proportion of respondents with at least one walking or cycling trip over the last week. The true prevalence, *p*, will always be less than the true participation, *π*, for *p* = *f* *π*, where 0 ≤ *f* ≤ 1, and *f* is the frequency of active travel. Since we have independent estimates for participation and prevalence, we can estimate the frequency and use it to estimate a weekly rate of physical activity due to travel, which is referred to here as *travel activity*.

The ratio of prevalence to participation may be used to estimate the frequency of active travel and is 0.286 and 0.264 in 2009 and 2017, respectively, or approximately once every 3–4 days.

### Regression models

We fit three multivariate regression models (prevalence, participation, and intensity) that include social and environmental variables, allowing us to estimate the effect of social variables after controlling for confounding due to environmental variables with strong effects. To represent different environments across the USA, we include variables that serve as surrogates for variations in infrastructure and city design (state), climate (state × season), access to businesses (population density) and access to personal automobiles (number of cars per household). We considered models for the *prevalence, participation,* and *intensity* and used the variables listed in the data description along with a state variable and a state by season cross-product. The state variable serves as a surrogate for differences in climate, infrastructure, and city design, while the state by season cross product accounts for seasonal differences. Subjects were classified as having engaged in active travel if they reported any walking or cycling on the survey day, *i.e.,* nonzero travel activity. We used logistic regression for both the prevalence and participation models.

*Travel activity* is defined as an individual’s amount of physical activity due to active travel, measured as a weekly rate in terms of MET-hours/week. An *active traveler* is defined as an individual with nonzero travel activity, and the *travel intensity* is a population-level measure defined as the mean of travel activity among active travelers ^11^. Travel intensity varies across social and environmental variables, albeit not as drastically as prevalence and participation. Travel activity is modeled using a log-normal distribution therefore we use the logarithm of travel activity in our regression models ^11^. Use of the state-level location also allows us to account for the over-sampling that occurred in thirteen of the states. Without the inclusion of a state variable some states would exert greater influence on the overall result than others, making for a poor representation of the whole population.

### Health estimates

We quantified differences in active travel among various US socioeconomic groups and the corresponding potential difference in all-cause mortality rates using the HOT model, a comparative risk assessment of a change in the distribution of travel activity ^11^. The HOT model uses the distribution of leisure time physical activity from Arem *et al.* (2015) and compares it to the distribution found by adding (or subtracting) the difference in travel activity between two populations ^17^. These two distributions, along with an exposure-response function for all-cause mortality also estimated from Arem *et al.*, make up a comparative risk assessment which allows the estimation of the population attributable fraction ^17^. The HOT model assumes that travel activity is distributed as a mixture of a log-normal distribution and a point-mass at zero and that all new travel activity is additional physical activity with no substitution from other domains ^11^. We tabulate these estimates in Table 2. For comparison across population groups, we set the baseline reference group as White, non-Hispanic, males, with a high school education, residing in California, in the fall, in a census tract with 2,000 to 3,999 people per square mile, with a household income of USD 35,000–49,999 and two cars available to the household.

**Table 2.** The HOT model parameters for eight US subpopulations for 2009 and 2017. *Participation* is the proportion who engage in active travel, *intensity* the strenuousness of the active travel, and frequency is the frequency of active travel among active travelers. *Overall Travel Activity (TA)* is the product of participation and intensity and represents the travel-related physical activity for the entire subpopulation. The reference group in both models is made up of White, non-Hispanic, male residents of California with a household income of USD 35,000–49,999, no more than a high school education, living in a census tract with population density of 2,000 to 3,999 people per sq. mi., two cars available to the household, and surveyed in the fall. Each of the seven non-reference subpopulations are created by changing one variable from the reference group. Δ *TA* is the change in TA from the reference subpopulation. PAF (population attributable fraction) is estimated using the HOT model and an exposure-response function for all-cause mortality and represents the proportional change in all-cause mortality.

	Participation		Intensity [MET-hours/ week]		Frequency		Overall TA [MET-hours/ week]		Δ TA (%)		PAF (%)	
	2009	2017	2009	2017	2009	2017	2009	2017	2009	2017	2009	2017
Reference population	0.65	0.65	2.48	2.63	0.20	0.17	1.62	1.70	NA	NA	NA	NA
Female	0.64	0.66	2.25	2.26	0.20	0.17	1.44	1.50	-10.80	-12.02	0.17	0.23
Asian	0.54	0.55	2.11	2.11	0.17	0.15	1.14	1.17	-29.47	-31.47	0.62	0.69
Black or African American	0.62	0.59	1.73	1.51	0.15	0.12	1.06	0.89	-34.18	-47.54	0.71	1.05
Hispanic	0.60	0.60	2.40	1.79	0.18	0.14	1.43	1.07	-11.32	-37.00	0.21	0.80
Graduate/Professional degree	0.78	0.82	3.69	4.01	0.29	0.25	2.88	3.28	78.49	92.67	-1.73	-1.87
Highest income bracket	0.70	0.75	3.20	3.55	0.24	0.20	2.24	2.66	38.79	56.06	-0.89	-1.16
Population density > 25,000	0.77	0.83	5.29	5.35	0.37	0.29	4.05	4.46	150.85	161.83	-2.91	-2.95
Zero cars in household	0.81	0.88	8.10	9.96	0.44	0.46	6.60	8.76	308.41	413.75	-5.07	-5.80

The prevalence and participation for each subgroup are found as *p _j_* = *e* ^*β*_0_ + *β _j_*^ and π _*j*_ = *e* ^*β*_0_ + *β _j_*^, where *β*_0_ and *β _j_* are the respective regression model coefficients. Daily intensity estimates on a log-scale are found as *μ _j_* = *β*_0_ + *β _j_* and σ _0_ is the residual standard deviation of the intensity model (log-scale). The transformation to a linear parameter space ( $\mu_{j}^{*}$ , $\sigma_{j}^{*}$ ) is done as follows.


$$\mu_{j}^{*}=e^{\mu_{j}+\frac{1}{2}\sigma_{0}^{2}}$$

$$\sigma_{j}^{*}=\sqrt{\left(e^{\sigma_{0}^{2}}-\;1)\;(e^{2\mu_{j}+\sigma_{0}^{2}}\right)}$$

The parameters are then scaled to represent a weekly rate ( $\mu_{j}^{'}$ , $\sigma_{j}^{'}$ ).


$$f_{j}=\frac{p_{j}}{\pi_{j}}$$

$$\mu_{j}^{'}=7f_{j}\mu_{j}^{*}$$

$$\sigma_{j}^{'}=7f_{j}\sigma_{j}^{*}$$

We simulate a vector of travel activity among the reference group, TA _0_, with length one million from a mixture of a log-Normal distribution and a point-mass at zero with parameters (π _0_, $\mu_{0}^{'}$ , $\sigma_{0}^{'}$ ). We do the same for each subgroup, TA _*j*_, using the parameters . The differences *δ _j_* = TA _*j*_ – TA _0_ are computed and added to a simulated vector of leisure time physical activity values, PA _0_, drawn from an empirical distribution estimated using data from Arem *et al*. ^18^ A function describing the empirical distribution of baseline leisure time physical activity is available and documented in the HOT R Package ^19^.


$$\text{P}{\text{A}}_{j}=\text{P}{\text{A}}_{0}+\delta_{j}$$

If negative, estimates for PA _*j*_ are set to zero. Vectors of 10,000 evenly spaced quantiles representing the distributions of PA _0_ and PA *_j_*, *q* ^0^ and *q ^j^*, are then used to estimate the population attributable fraction, *ρ _j_*, for each subgroup j relative to the reference group.


$$\rho_{j}=\frac{\sum_{l=1}^{m}R(q_{l}^{j})}{\sum_{l=1}^{m}R(q_{l}^{0})}-1$$

The function *R* is an exposure-response function for all-cause mortality and leisure time physical activity given in terms of MET-hours/week. The exposure-response function used in this analysis is a piecewise linear function created from hazard ratios found in Arem *et al.* ^18^ This exposure-response function, along with others, is available and documented in the HOT R package ^20^.

All results were computed using the R programming language and environment for statistical computing version 4.2.1 ^21^. All multivariate regression models were carried out using *glm* methods in the R core package *stats* and nominal significance is set at the *α* = 0.05 level. The R package *HOT* contains many of the functions used in the analysis and is available on GitLab ^20^. The analysis here is a secondary analysis of a publicly available database with no identifiable data. As such, IRB approval of informed consent requirement by an IRB is waived. 

## Results

The sample size and proportion of each of the model variables are displayed in Table 1 in which we see the following proportions (expressed as a percentage) by race; American Indian or Alaska Native (0.8, 0.5), Asian (4.5, 8.8), Black or African American (6.6, 8.4), Multiple Responses Selected (0.9, 3.1), Native Hawaiian or other Pacific Islander (0.5, 0.3), Other (6.2, 3.3), White (80, 76). “I don’t know” and refusal to answer were recorded as NA (1.1, 0.6).

### Active travel prevalence and participation

**Figure 1.**
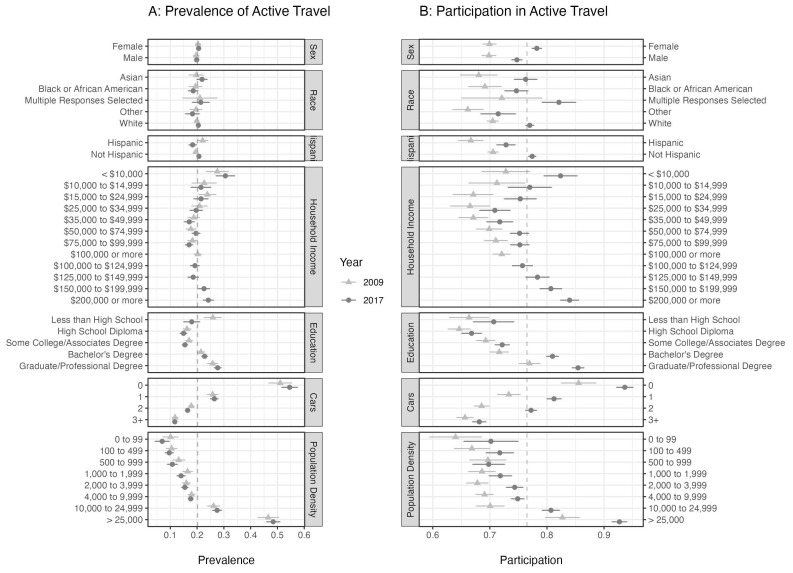
The prevalence (Panel **A**) and participation (Panel **B**) estimates (using survey weights) with 95% confidence intervals for adults living in US metropolitan areas with a population size greater than one million. The dashed, vertical lines are drawn at the overall mean values. Prevalence is a daily rate of active travel, while the participation is a weekly rate. The prevalence was estimated by counting the number of individuals who recorded a trip by walking or cycling on the survey day. Participation is estimated as the proportion of people who reported having taken at least one walk or cycling trip in the last week. Estimates for *American Indian or Alaskan Native*, and *Native Hawaiian or other Pacific Islander* are not displayed here due to small sample sizes (< 1% of total sample).

The prevalence and participation of active travel were estimated across each of the variables independently (weighted estimates) and are shown in Figure 1 with 95% confidence intervals. The overall prevalence was 20.0% (19.3%, 20.8%) and 20.2% (19.6%, 20.8%) in 2009 and 2017, respectively. The overall participation was 69.9% (69.0%, 70.7%) and 76.5% (75.9%, 77.1%) in 2009 and 2017, respectively. A shift in participation values across all groups was observed from 2009 to 2017 with the overall mean increasing by 6.6%. The increase in values for participation did not affect the pattern for any of the model variables, except sex in which we see that in 2017 the participation was greater among females than males, while in 2009 they were equal. 

 Using the R package *survey* version 4.1-1, we computed Pearson chi-squared statistics (adjusted by design effect) using the survey design weights and found that both the prevalence and participation of active travel for each variable (tested independently) yielded highly significant results, with only the sex and race variables not significant at the *α* = 0.05 level in all four models (2009, 2017 × prevalence, participation) ^22^. Table 3 contains log transformed p-values (–log _10_ *⁡p*) for all tests and in it we see that sex is highly significant in the 2017 participation model (–log _10_ p = 7.15), and race is marginally significant in the 2009 model (–log _10_⁡ p = 1.38), and not significant at all in the 2017 prevalence model (–log _10_ *p* = 0.87). In both 2009 and 2017, the estimates for prevalence among the Black or African American population were not significantly different than the overall mean ( Figure 1A). The prevalence in the Hispanic population changed dramatically from 2009 to 2017, going from greater than the non-Hispanic population to less than ( Figure 1A). With household income, we observe similar patterns in the prevalence and participation models in 2009 and 2017. Using the participation measure we see a U-shaped relationship across household income with the minimum at USD 25,000–34,999. With the prevalence measure, however, the location of the minimum is not clear. Prevalence is greatest at the lowest household income level. For all education levels above *Less than High School* we see an increasing trend in both prevalence and participation with above-average values for *Bachelor’s Degree* and above. There was no significant difference in prevalence across sex.

**Table 3.** -log _10_ transformed *p*-values from a weighted chi-squared test of association for survey data using the R package *survey* and function *svychisq*.

	Prevalence		Participation	
Variable	2009	2017	2009	2017
sex	0.42	0.62	0.04	7.15
race	2.93	0.87	1.38	4.95
hispanic	1.65	2.30	3.20	6.81
education	17.25	54.83	17.10	93.61
income	4.44	13.88	2.49	14.95
ncar	125.01	290.26	24.39	92.68
pop.density	114.77	270.69	17.09	70.80
nhouse	8.62	49.67	11.74	74.85

Note that –log _10_ 0.05 = 1.3.

### Prevalence and participation logistic regression

We see in Figure 1 that the variables with the largest effect size are environmental, namely population density and number of cars per household. Thus, to truly understand the effect of the social variables we must use a model that accounts for all the variables simultaneously. We constructed three multivariate logistic models using the variables described above (sex, age, population density, income, education, number of cars, race, Hispanic status, race × Hispanic status, state × season). In the prevalence model, the response variable was *I*_*TA* > 0_, and in the participation model *I*_*nwalk*+ *ncycle* > 0_, where *TA* is travel activity and *nwalk* and *ncycle* are the self-reported number of walking and cycling trips taken over the last week. 

**Figure 2.**
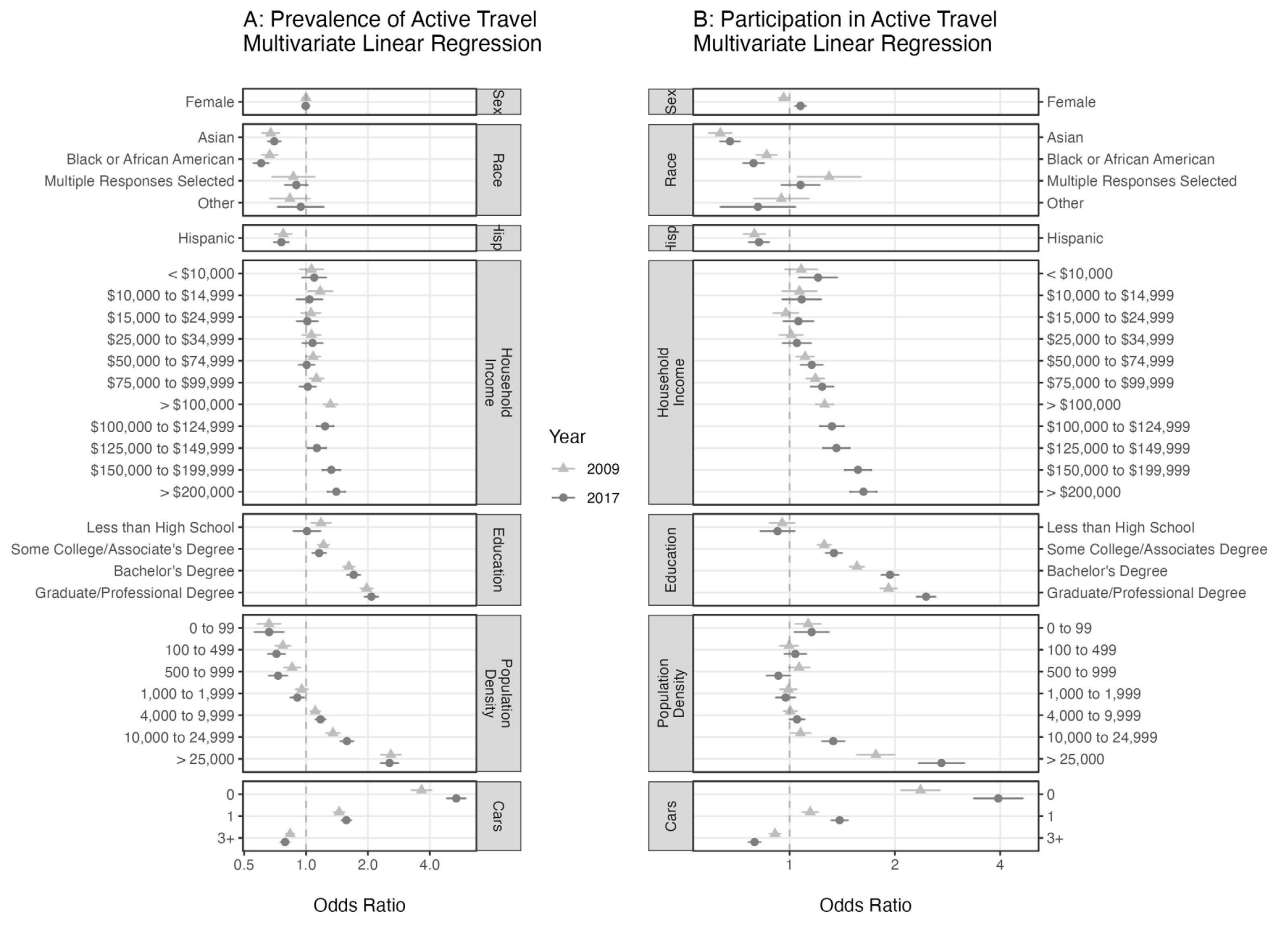
The odds ratios from two multivariate logistic regression models. The odds ratio for daily active travel is presented with 95% confidence intervals in Panel A. Panel B is the participation model in which the odds ratios of weekly active travel are presented. Variables for age, state and state by season were included in both models to account for seasonal effects and over-sampling of some states and are not displayed here. The reference group in both models is made up of White, non-Hispanic, male residents of California with a household income of USD 35,000-49,999, a high school education, living in a census tract with population density of 2,000 to 3,999 people per square mile, two cars available to the household, and surveyed in the fall.

Figure 2 displays the results of the prevalence and participation regression models for all variables except state, state by season, and race by Hispanic status. While the variables with the greatest effect size are still environmental variables (population density and number of cars), in the multivariate regression models we observe significant, consistent effects on both prevalence and participation due to race, in particular *Black or African American* and *Asian*, that are not as clear in the univariate analyses of Figure 1. Consistent trends are observed in both 2009 and 2017 in which the odds of active travel increase as the population density of the household’s census block increases and as the number of cars per household decreases. In 2017, an individual living in a census block with population density greater than 25,000 people per square mile is more than twice as likely to engage in active travel as the same person living in a census block with a population density of 2,000 to 3,999 (reference group). Similarly, in 2017 someone living in a household with no cars is more than four times as likely to engage in active travel daily (prevalence) compared to a person with the same characteristics except living in a house with two cars (reference group). 

Among the social variables, education has the largest effect size, increasing active travel as education level increases. A person with a graduate or professional degree is approximately twice as likely to engage in active travel than a person with all the same characteristics except having only a high school education. Prevalence and participation across household income, unlike the other ordered variables, do not strictly increase or decrease. A clear pattern appears in the participation model in which the minimum odds ratios occur in the middle of the range at USD 25,000–34,999. The upper and lower ends, however, are not equal. High-income households have odds greater than low-income households. A similar pattern is observed in the prevalence model, albeit without much statistical significance at the low end. The Black or African American and Asian populations showed significantly lower adjusted odds of active travel in both the prevalence and participation models in 2009 and 2017 compared to the reference group (White). In the participation model, we estimate the odds ratio for *Black or African American* to be 0.860 ( *p* = 8.4 × 10 ^–6^) and 0.788 ( *p* = 8.6 × 10 ^–12^), and for *Asian* 0.634 ( *p* = 4.3 × 10 ^–32^) and 0.675 ( *p* = 7.9 × 10 ^–31^), for 2009 and 2017 respectively. In 2017, the odds of participation were slightly greater in the female population than in the male population (OR = 1.07, *p* = 1.1 × 10 ^–4^). Previously, however, in 2009 the direction was opposite with the odds of active travel slightly less among females than males (OR = 0.962, p = 0.02). 

### Travel intensity linear regression

**Figure 3.**
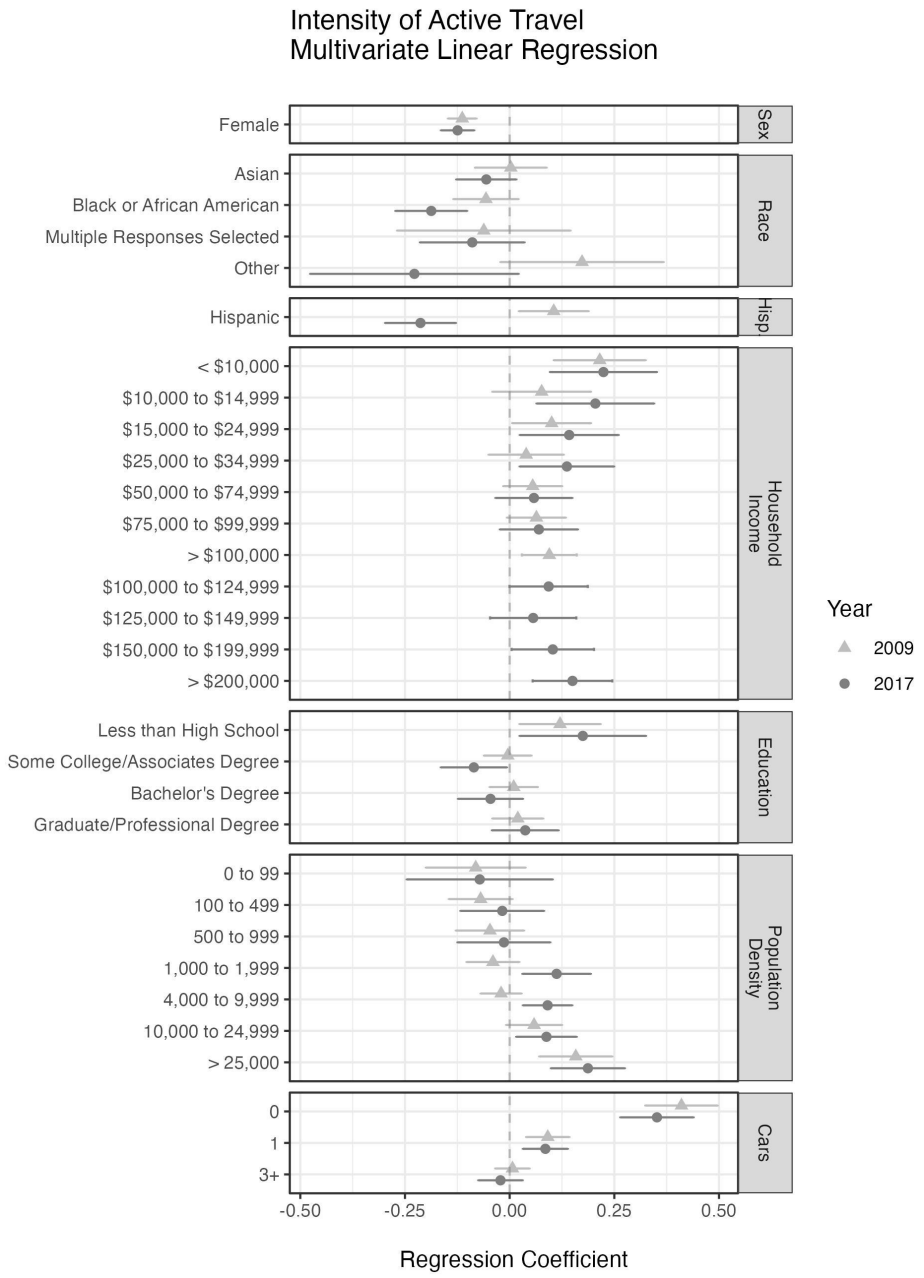
The regression coefficients from a multivariate linear regression model using the logarithm of travel activity among active travelers. The coefficients are presented with 95% confidence intervals. Variables for age, state, and state by season were included to account for seasonal effects and over-sampling of some states and are not displayed here. The reference group in both models is made up of White, non-Hispanic, male residents of California with a household income of USD 35,000-49,999, no more than a high school education, living in a census tract with population density of 2,000 to 3,999 people per square mile, two cars available to the household, and surveyed in the fall. American Indian, Alaskan Native, Native Hawaiian, or other Pacific Islander were not included due to small sample size (less than 1%).

A forest plot of regression coefficient estimates for the travel intensity model is presented in Figure 3. In it we see that in both 2009 and 2017, the female population showed less intensity (regression coefficient *β* = –0.113 and –0.124, with *p* = 9.8 × 10 ^–11^ and 1.0 × 10 ^–9^, in 2009 and 2017) of active travel than their male counterparts. In 2017 the *Black or African-American* population also showed less intense travel activity ( *β* = –0.187, *p* = 1.87 × 10 ^–5^) than their *White* counterparts, however in 2009 the effect was not significant. In 2017 the *Hispanic* population showed less intensity ( *β* = –0.213, *p* = 8.13 × 10 ^–7^) of active travel than *non-Hispanic* individuals, but in 2009 the effect for intensity was reverse with *Hispanic* greater than *non-Hispanic* ( *β* = 0.105, *p* = 0.013). The lowest level of education was the only education group that demonstrated a significant increase in intensity ( *β*_2009_ = 0.175, *p* = 0.0234 and *β*_2017_ = 0.120, *p* = 0.0149) while all other levels remained near the mean. As with prevalence and participation, intensity increases as the population density or the number of cars per household decreases. The pattern of travel intensity across the household income variable is similar to the ones seen in the participation and prevalence models where the low and high ends show a significant positive effect, and the middle-income levels are not significantly different than the referent group (USD 35,000–49,999). Note that a daily rate for travel activity was used in the regression analyses and later scaled to a weekly rate.

### Health estimates

The mean overall travel activity in a population is the product of participation and intensity. Using the multivariate regression models, we see that participation in the reference group is 0.650 and 0.648, and the intensity is 2.484 and 2.629 MET-hours/week in 2009 and 2017, respectively. The overall mean travel activity in the reference group is therefore 1.615 and 1.704 MET-hours/week. For both 2009 and 2017 changing the sex variable to female in the model yields a reduction of 1.44 and 1.50 MET-hours/week, a decrease of 10.8% and 12.0% compared to the male reference group. Table 2 presents the overall mean travel activity and percent change compared to the reference group for several populations. The Black or African American reference population has expected travel activity lower than the White reference group by 34.2% and 47.5% in 2009 and 2017, respectively. Similarly, the Asian population has lower expected travel activity by 29.5% and 31.5% in 2009 and 2017 compared to the White reference group. The highest education level shows significantly greater travel activity than the *High School Diploma* (reference) in both 2009 and 2017 with a 78.5% and 92.7% increase, respectively. Using the HOT model, we estimate the population attributable fraction for all-cause mortality associated with these reductions or increases in participation and/or intensity. A large reduction in active travel, as in the Black or African American population compared to the White population, may be sufficient to increase the all-cause mortality rate by almost one percent (0.7% and 1.1% in 2009 and 2017, respectively). See Table 2 for health estimates for the *Hispanic* and *Asian* variables and others. Since the estimated number of deaths from non-communicable disease among adults aged 20–65 in US metropolitan areas with a population greater than one million is 274,313 and 311,789 deaths per year in 2009 and 2017 ^23, 24^, a population attributable fraction of 1%, if applied to the entire population, is equivalent to 2743 and 3118 deaths averted in 2009 and 2017 ^23, 24^. Based on our models, the difference in active travel between those with a *High School Diploma* and those with a *Graduate or Professional Degree* corresponds to a 1.7% and 1.9% percent decrease in mortality or, if applied to the entire population, the equivalent of 4,746 and 5,830 premature deaths averted in 2009 and 2017, respectively.

## Discussion

Using odds ratios for active travel in the USA that are adjusted for multiple social and environmental variables, we observed that racial and ethnic minority populations (Black or African American, Asian, and Hispanic) are less likely to engage in active travel than the White population with the same socioeconomic and environmental characteristics in both 2009 and 2017. The most influential factors in active travel are environmental, *e.g.*, population density and access to personal automobiles, but sociodemographic variables such as race, income, and education are also significant. This disparity in active travel creates a potential health burden in some populations which we estimate may be responsible for a relative increase in all-cause mortality of approximately one percent.

This analysis is the first to demonstrate that the adjusted odds for active travel among the Black or African American and Asian adult populations in US major metropolitan areas are less than their White counterparts. In contrast to this study, previous studies of US transportation surveys considered the entire US and reported that walking and active travel are both more prevalent among minority populations than the White population ^12, 25, 26^. An analysis by Paul *et al.* of transportation-related walking reported odds ratios adjusted for sex, age, race, education and BMI and observed that the adjusted odds of walking for transportation was highest among the non-Hispanic Black population, that the prevalence increased with increasing education level, and that the prevalence of walking for transportation was lowest in the South ^27^. We chose to restrict the population to major metropolitan areas to remove some of the confounding between the race and walking relationship due to urbanicity and population density.

Whitfield *et al.* reported that members of minority populations were more likely to walk for transportation than members of the non-Hispanic White population during 1999–2012 ^12^. Another study reported that the prevalence of walking 30 minutes per day was higher in the Hispanic, African-American and Asian populations than in the White population ^25^. It was estimated that in 2005 the highest prevalence of transportation walking in the USA was among non-Hispanic Black men (36.0%) and Asian/Native Hawaiian/Pacific Islander women (40.5%) ^26^.

The disparity in cycling prevalence across race is much greater than the disparity in walking, with the odds of cycling being significantly lower in minority populations. There are several potential explanations for these observed results. Exclusionary zoning, discrimination, systematic and institutional racism have all contributed to the inequities in minority and low-income communities across the USA resulting in less access to safe street infrastructure and green space ^28– 32^. Racial profiling, harassment, and discriminatory treatment, along with a lack of access to cycling and educational resources, can discourage low-income communities and communities of color from cycling ^32, 33^. For racial and ethnic minorities and those living in low-income communities, concerns about personal safety due to traffic collisions and crime are two of the top-cited barriers to engaging in active travel modes such as cycling ^28, 29^.

Researchers have found that walking due to concerns about crime is significantly larger among women than men and any attempt to improve walkability must address, in particular, the safety of women ^30^. Moreover, concerns about gentrification may limit investments in walking and cycling infrastructure, given that improvements in the built environment have been associated with increasing property and housing values thereby posing a risk to long-term residents who have to contend with the tensions of revitalization and displacement ^31^.

Equity is an important consideration in the development and implementation of policies, plans, initiatives, and programs designed to improve health and well-being by increasing participation in active travel. Planning to improve walking and cycling infrastructure and creation of policies, initiatives, plans, and programs to increase access to active travel, must be informed by key environmental, socioeconomic and demographic factors that drive existing inequities ^32, 33^. Equitable approaches are especially warranted as our study finds significant difference in the adjusted odds of active travel between the Black or African American and Asian populations compared to the White population. Future studies should investigate the specific drivers of these disparities in metropolitan areas, including the extent of disparities in walking and cycling infrastructure across metropolitan areas ^29^. Improving representation in the decision-making process and targeted outreach to transportation in disadvantaged groups and communities throughout the planning and decision-making process will be essential for increasing equity in active travel and in the resulting health benefits ^33^.

### Limitations

 Assumptions regarding the distribution and replacement of physical activity are necessary and therefore the estimates for health benefits are broad. The trends across the model variables were remarkably consistent between 2009 and 2017, however we did observe an increase in participation estimates across all variables in 2017. Changes in survey methodology between the 2009 and 2017 survey likely affected our results. The increase in participation from 2009 to 2017 was likely due to a questionnaire change. As of 2017, the questionnaire now explicitly prompts respondents to recall walk and bike trips ^34^. Additionally, walk and bike trips to and from home (loop trips) are included in 2017 but not in 2009 ^34^. The increase in participation estimates (while not also in prevalence estimates) will have the effect of decreasing the estimate for travel activity and intensity, due to the decrease in the frequency of active travel. Of note, the categorization of Hispanic/Mexican also changed between 2009 and 2017, therefore the insights that can be drawn in this population over time are limited in this study. As racial/ethnic disparities remain a clear concern when it comes to active transportation, physical activity and chronic illness, we need more precise and inclusive measures of racial and ethnic identity categories to be able to draw accurate conclusions.

 To arrive at estimates of the health impacts we must make assumptions regarding the underlying distribution of overall physical activity. If the underlying distribution of physical activity is already high in a population, changes to travel activity will have less of an effect. Since it is unknown how this distribution varies across the social and environmental variables considered here, we assume that the distribution of physical activity is the same in all subpopulations.

The NHTS dataset has geographic resolution at the state level, giving the metropolitan area only if the population size is greater than one million. Thus, very little information can be included in the model to account for local urban design and culture. In future studies, we would like to include local measures of safety and walkability. Furthermore, capturing the lived experience of community members through qualitative methods, such as surveys, may help elucidate barriers to walking and biking, and may shed light on other ways that people that report low physical activity may increase their physical activity as part of their daily routine. Policy measures should focus on addressing structural factors that prevent people from active transport to reduce sociodemographic disparities. Efforts to design and evaluate interventions such as adult cycling lessons and walking groups can encourage more people to enjoy the health benefits of physical movement.

## Data Availability

Data used in this study are from the 2009 and 2017 U.S. National Household Travel Survey (NHTS) datasets, available from the NHTS website at https://nhts.ornl.gov/downloads. The analysis code is available at: https://gitlab.com/GHI-UW/nhts See tag named *release* branch for the state of the repository at the time of publication. Archived source code at time of publication: https://doi.org/10.5281/zenodo.7802854 ^35^. License: Creative Commons Attribution 4.0 International The R package HOT is available at: https://gitlab.com/GHI-UW/hot See tag named *release* for the state of the repository at the time of publication. Archived source code at time of publication: https://doi.org/10.5281/zenodo.7813760 ^20^. Licenses: Creative Commons Attribution 4.0 International and GNU General Public License, version 3
