# Supplementary material for: The influence of socioeconomic characteristics on active travel in US metropolitan areas and the contribution to health inequity
Source: Wellcome Open Res. 2024 Aug 5;8:266. Originally published 2023 Jun 21. [Version 2] doi: 10.12688/wellcomeopenres.19147.2 (PMC11425035; doi:10.12688/wellcomeopenres.19147.2)
Supplement: Peer review history

# Reviewer report: Reviewer response for version 2

**DOI:** 10.21956/wellcomeopenres.25092.r92164

**Published:** 2024-09-26

**Authors:** Jesus Barajas

**Affiliations:** 1 Department of Environmental Science and Policy, University of California Davis, Davis, California, USA

The authors have satisfied my initial concerns. I have no further comments.

Is the work clearly and accurately presented and does it cite the current literature?

Partly

If applicable, is the statistical analysis and its interpretation appropriate?

Yes

Are all the source data underlying the results available to ensure full reproducibility?

Yes

Is the study design appropriate and is the work technically sound?

Yes

Are the conclusions drawn adequately supported by the results?

Yes

Are sufficient details of methods and analysis provided to allow replication by others?

Partly

Reviewer Expertise:

Transportation planning, equity and justice, travel behavior

I confirm that I have read this submission and believe that I have an appropriate level of expertise to confirm that it is of an acceptable scientific standard.

# Reviewer report: Reviewer response for version 2

**DOI:** 10.21956/wellcomeopenres.25092.r95785

**Published:** 2024-09-25

**Authors:** Jamie Furlong

**Affiliations:** 1 Westminster University, London, England, UK

This is an interesting piece of work that I enjoyed reading. It provides a detailed account of the individual- and area-level predictors of walking and cycling in the US context. It is concise and generally easy to read. 

 There are, however, some small additions/suggested changes: 

1. There is some missing literature - the authors give the impression little is known about the sociodemographic/area level predictors of active travel in the US. But there is some literature that should be cited. For example: ' Tribby and Tharp (2019 ^1^) Examining urban and rural bicycling in the United States: Early findings from the 2017 National Household Travel Survey'. Also, Riggs and Sethi (2019 ^2^) 'Multimodal travel behaviour, walkability indices, and social mobility: how neighbourhood walkability, income and household characteristics guide walking, biking & transit decisions'. 
2. In the discussion, can you reflect a little on the implications of the density finding? It may be a more obvious finding but the paper could serve as a call for specific policies to increase population density in cities across the US. You could note that it adds to a significant body of research calling for higher density development and link to concepts like 15-minute cities etc. 
3. A few sentences need re-writing/amending: 

- 'faced by the United States' reads a bit strangely 
- bold font for Prevalence and participation logistic regression
- 'Researchers have found that walking due to concerns about crime is significantly larger among women than men' - not sure I understand this sentence
- 'p =' inconsistency in italicised or not. 

I'm happy to recommend that, with these minor changes, this article is indexed.

Is the work clearly and accurately presented and does it cite the current literature?

Partly

If applicable, is the statistical analysis and its interpretation appropriate?

Yes

Are all the source data underlying the results available to ensure full reproducibility?

Yes

Is the study design appropriate and is the work technically sound?

Yes

Are the conclusions drawn adequately supported by the results?

Yes

Are sufficient details of methods and analysis provided to allow replication by others?

Yes

Reviewer Expertise:

Active travel interventions, spatial analysis, longitudinal survey data

I confirm that I have read this submission and believe that I have an appropriate level of expertise to confirm that it is of an acceptable scientific standard.

## References

[1] : Examining urban and rural bicycling in the United States: Early findings from the 2017 National Household Travel Survey. *Journal of Transport & Health* .2019;13: 10.1016/j.jth.2019.03.015 143-149 10.1016/j.jth.2019.03.015

[2] : Multimodal travel behaviour, walkability indices, and social mobility: how neighbourhood walkability, income and household characteristics guide walking, biking & transit decisions. *Local Environment* .2020;25(1) : 10.1080/13549839.2019.1698529 57-68 10.1080/13549839.2019.1698529

# Reviewer report: Reviewer response for version 1

**DOI:** 10.21956/wellcomeopenres.21225.r70251

**Published:** 2024-05-14

**Authors:** Jesus Barajas

**Affiliations:** 1 Department of Environmental Science and Policy, University of California Davis, Davis, California, USA

* Is the work clearly and accurately presented and does it engage with the current literature?

 In general, the work is clearly presented. The writing is clear and concise and presents the results in a straightforward manner. The equations and descriptions of the parameter estimates in the Health Estimates section is a little challenging for those who generally do not read detailed statistical methods, but it describes the methods precisely. The work engages briefly with the current literature. In the introduction, the authors write: "...and one of the few to use regression models with social and environmental covariates." This statement doesn't strike me as true. Even a quick search on Google Scholar brings up several relevant studies on the first page that use the NHTS to estimate active travel while adjusting for socioeconomic and neighborhood factors. I suggest removing or qualifying this statement.

 * Is the study design appropriate and does the work have academic merit?

 The study design is mostly appropriate. One challenge I have with the work is that the estimates for physical activity assume the same distribution across the population. The authors address this as a limitation in the final section. However, We have some evidence from research using the American Time Use Survey on how physical activity differs across socioeconomic characteristics. See Saffer et al. (2013) [Ref-1]. Because the HOT model relies on the underlying distribution of leisure time physical activity, it's an important omission from the research when quantifying active travel's contribution to all-cause mortality. I suggest the authors revisit this assumption and make reasonable adjustments to the distribution of physical activity based on the literature.

 A second point relates to the removal of records for exercise trips. Trips with a purpose of exercise were removed from the dataset because they were "unrelated to travel." However, this is not strictly true because the variable is a bit messy. It includes walk trips like walking for exercise, but it would also include walk trips to get to a gym for other exercise. That second walk trip would meet the definition of active travel. I don't agree that these trips should be removed.

 * Are sufficient details of methods and analysis provided to allow replication by others?

 The details of model and analytical specifications are provided. A fuller description of the HOT model is warranted. While the authors point to a reference where the model was first described and used, I found it confusing to flip back and forth between the two articles to understand how the model is contributing to the methodological framework. 

 * If applicable, is the statistical analysis and its interpretation appropriate?

 The statistical analysis and interpretation appear to be appropriate.

 * Are all the source data underlying the results available to ensure full reproducibility?

 The authors provide an R repository that contains the code necessary to reproduce the results.

 * Are the conclusions drawn adequately supported by the results?

 The conclusions are supported by the results. The authors should endeavor to explain in more detail why their results differ from previous studies--in particular, why certain socioeconomically disadvantaged groups are shown to walk and cycle less and not more than others.

Is the work clearly and accurately presented and does it cite the current literature?

Partly

If applicable, is the statistical analysis and its interpretation appropriate?

Yes

Are all the source data underlying the results available to ensure full reproducibility?

Yes

Is the study design appropriate and is the work technically sound?

Yes

Are the conclusions drawn adequately supported by the results?

Yes

Are sufficient details of methods and analysis provided to allow replication by others?

Partly

Reviewer Expertise:

Transportation planning, equity and justice, travel behavior

I confirm that I have read this submission and believe that I have an appropriate level of expertise to confirm that it is of an acceptable scientific standard, however I have significant reservations, as outlined above.

## References

[1] : Racial, Ethnic, and Gender Differences in Physical Activity. *J Hum Cap* .2013;7(4) : 10.1086/671200 378-410 10.1086/671200 25632311 PMC4306423

# Response

**Published:** 2024-07-02

**Authors:** Samuel Younkin

**Affiliations:** University of Wisconsin-Madison, USA

We thank the reviewer for their comment about the existence of comparable studies that also adjust for socioeconomic variables. To address this we have made the following change to line 26. "Our analysis is the first to restrict the sample to major metropolitan areas, and one of the few to use regression models with social and environmental covariates." was changed to "Our analysis, performed using regression models that include social and environmental covariates, is the first to restrict the sample to major metropolitan areas."

 Thank you for bringing attention to the choice for the distribution of background physical activity. The end of the second paragraph in the Limitations section states, "Since it is unknown how this distribution varies across the social and environmental variables considered here, we assume that the distribution of physical activity is the same in all subpopulations." This is not precisely correct. The crux of the problem is that our comparison is between the distribution of physical activity in the reference population and the same distribution shifted by the change in travel activity that results from changing one variable in the regression models. The primary limitation is that the methodology is not symmetric with respect to choice of reference population and the distribution chosen to represent the reference population cannot be specified precisely. For consistency with our choice for the exposure-response function we use the sample statistics from Arem et al. to approximate the baseline distribution. To address this we replaced paragraph 2 of the Limitations subsection. "To arrive at estimates of the health impacts we must make assumptions regarding the underlying distribution of overall physical activity. If the underlying distribution of physical activity is already high in a population, changes to travel activity will have less of an effect. Since it is unknown how this distribution varies across the social and environmental variables considered here, we assume that the distribution of physical activity is the same in all subpopulations." has been changed to "To make estimates about the effect of model variables on all-cause mortality we must use a hypothetical reference population, assume a distribution of physical activity for that population, and compare that against the distribution found by adding the change in physical activity due to active travel estimated from the regression coefficients described above. The necessity of specifying the baseline distribution is indeed a limitation for physical activity is known to vary across demographic variables such as race and sex 37. It is important to remember that the results presented here must be viewed as the influence a given model variable has on a hypothetical population in which the distribution of physical activity is that of the sample in the study by Arem et al.18" where the citation 37 is for Saffer et al. (2013).

 We agree with the reviewer that including trips with the purpose of "Go to gym/exercise/play sports" improves the analysis. Line 42 of WOR-1-2009.Rmd and line 41 of WOR-1-2017.Rmd were edited so that no trips are excluded based on the WHYTO variable. The statement on lines 82-85: "Trips in the 2017 survey with the stated purpose Exercise (go for a jog, walk, walk the dog, go to the gym), and trips in the 2009 survey with the purpose Go to gym/exercise/play sports were removed from the analysis (3.4% and 3.9% of all trips, respectively) since unrelated to travel." was removed from text. All figures, tables, and results were updated after re-running analysis. We agree with the reviewer that the HOT model needed a more fuller description. We have replaced lines 132-140: "The HOT model uses the distribution of leisure time physical activity from Arem et al. (2015) and compares it to the distribution found by adding (or subtracting) the difference in travel activity between two populations 17 . These two distributions, along with an exposure-response function for all-cause mortality also estimated from Arem et al., make up a comparative risk assessment which allows the estimation of the population attributable fraction 17 . The HOT model assumes that travel activity is distributed as a mixture of a log-normal distribution and a point-mass at zero and that all new travel activity is additional physical activity with no substitution from other domains." with a more lengthy description of the HOT model. See revised manuscript for text. (Note that the equations and length of the fuller description make it difficult to post here.)

 Thank you for the comment regarding results that differed from prior studies. We have provided more detail through an update of the Discussion section as follows: -

 First sentence of Paragraph 2

   - we have changed "is the first" to "is among the first."

 Second sentence of Paragraph 2

   - we have changed "In contrast to this study, previous..." to "Previously..."

 Fourth sentence of Paragraph 2

   - we have removed "We chose to restrict the population to major metropolitan areas to remove some of the confounding between the race and walking relationship due to urbanicity and population density."

 Paragraph 3

   - Inserted at the end: "Our results differed from the previously mentioned studies by Whitfield, Pucher, and Kruger because those studies neither included urban/rural status nor subset the sample to include only residents of major metropolitan areas 12,

 25, 26. Correlation between race and residence in rural areas, along with low rates of active travel in rural settings, confounds the relationship between active travel and race. To avoid this, we chose to subset the sample based on residence in a major metropolitan area. In a more recent study Buehler et al. did include urban/rural status in their statistical analysis of walking and cycling in the U.S. and identified an increase in the odds ratio for both walking and cycling among the Non-Hispanic White population, relative to all other populations 36."

 Note that a new reference to Buehler is included in the above paragraph.

# Reviewer report: Reviewer response for version 1

**DOI:** 10.21956/wellcomeopenres.21225.r70248

**Published:** 2023-12-28

**Authors:** Janet Wojcik

**Affiliations:** 1 College of Education, Sport, and Human Sciences, Winthrop University, Rock Hill, South Carolina, USA

Overall, the authors have done a good job analyzing the NHTS data from 2009 and 2017. There is great detail about their analyses and modeling in the article. 

 Only suggestions for the discussion/conclusions is perhaps indicate if the particular modeling has any influence on the outcomes of the study, that White persons had more active travel than Black, Asian, or Hispanic persons, or is it from the way the data are captured in the NHTS. 

 The NHTS 2022 data have been released and there were apparently changes to the survey which I am not sure if any comparisons can continue to be made from previous surveys. The 2009 and the 2017 surveys were similar. The 2022 survey does include more modes of transportation.

Is the work clearly and accurately presented and does it cite the current literature?

Yes

If applicable, is the statistical analysis and its interpretation appropriate?

Yes

Are all the source data underlying the results available to ensure full reproducibility?

Yes

Is the study design appropriate and is the work technically sound?

Yes

Are the conclusions drawn adequately supported by the results?

Yes

Are sufficient details of methods and analysis provided to allow replication by others?

Yes

Reviewer Expertise:

walkability, bikeability, active transportation, physical activity, exercise programs for special populations.

I confirm that I have read this submission and believe that I have an appropriate level of expertise to confirm that it is of an acceptable scientific standard.
